# Use of non-conventional biomarkers in the early diagnosis of acute kidney injury in preterm newborns with sepsis

**DOI:** 10.1590/2175-8239-JBN-2020-0222

**Published:** 2021-11-29

**Authors:** Joycilene da Silva Barbosa, Geraldo Bezerra da Silva, Gdayllon Cavalcante Meneses, Alice Maria Costa Martins, Elizabeth De Francesco Daher, Rosângela Pinheiro Gonçalves Machado, Romélia Pinheiro Gonçalves Lemes

**Affiliations:** 1 Universidade Federal do Ceará, Programa de Pós-Graduação em Patologia, Fortaleza, CE, Brasil.; 2 Universidade de Fortaleza, Centro de Ciências da Saúde, Faculdade de Medicina, Programa de Pós-Graduação em Saúde Pública, Fortaleza, CE, Brasil.; 3 Universidade Federal do Ceará, Faculdade de Farmácia, Departamento de Análises Clínicas e Toxicológicas, Fortaleza, CE, Brasil.; 4 Universidade de Fortaleza, Centro de Ciências da Saúde, Faculdade de Medicina, Fortaleza, CE, Brasil.

**Keywords:** Acute Kidney Injury, Sepsis, Infant, Premature, Biomarkers, Injúria Renal Aguda, Sepse, Recém-Nascido Prematuro, Biomarcadores

## Abstract

Acute kidney injury (AKI) is a common finding in Neotatal Intensive Care Units (NICU). Sepsis is one the main causes of AKI in preterm newborns. AKI has been associated with significant death rates. Early detection of the condition is the first step to improving prevention, treatment, and outcomes, while decreasing length of hospitalization, care costs, and morbimortality. AKI may progress to chronic kidney disease (CKD), a condition linked with dialysis and greater risk of cardiovascular disease. This review article aims to discuss cases of AKI in preterm newborns with sepsis, the use of biomarkers in lab workup, and the use of non-conventional biomarkers for the early identification of AKI.

## Resumo

A lesão renal aguda (LRA) é comum na Unidade de Terapia Intensiva Neonatal (nUTI) e a sepse é uma de suas principais causas, especialmente em prematuros. Apresenta altas taxas de mortalidade e sua detecção precoce é o primeiro passo para a prevenção dessa condição, pois permite o tratamento adequado e melhora o desfecho, diminui o tempo de internação, os custos não médicos e a morbimortalidade. Destaca-se ainda que a LRA pode evoluir para doença renal crônica (DRC), havendo a necessidade de diálise, com maior risco de desenvolver doenças cardiovasculares. Este artigo de revisão tem como objetivo discutir a LRA em recém-nascidos (RNs) prematuros com sepse, abordando biomarcadores utilizados na rotina laboratorial e principalmente a utilização de biomarcadores não tradicionais para identificação precoce de LRA.

## Introduction

Acute kidney injury (AKI) is defined as the sudden impairment of kidney function followed by acute reversible increases in serum creatinine (SCr) associated or not with decreased urine output, resulting in inability to maintain proper homeostase of fluids, electrolytes, and residues. It is a complex multifactorial disorder that ranges from mild injury to kidney failure, for which renal replacement therapy may be required^1^^,^ 2.

AKI may be categorized as follows: (i) prerenal AKI, a condition caused by hypovolemia, renal artery contraction or vasodilation that may produce glomerular perfusion impairment secondary to decreased circulatory volume without renal alterations; (ii) renal AKI, a condition caused by all other types of kidney disease, including capillary and glomerular disease, vascular kidney diseases, acute interstitial nephritis, and acute tubular necrosis; and (iii) postrenal AKI, a condition seen in cases of acute urinary obstruction^3^^,^ 4.

The causes of AKI in newborns include very low birth weight (bodyweight of less than 1,500 g), respiratory distress syndrome, a low 5-minute Apgar score, intubation at birth, cardiac arrest, and use of medication^5^^,^ 6.

In terms of risk subgroups, preterm newborns (with a gestational age [GA] of less than 37 weeks) may be categorized as follows: extremely preterm - babies born before 28 weeks of pregnancy; very preterm - babies born between 28 weeks and 31 weeks and 6 days of pregnancy; moderate preterm - babies born between 32 weeks and 33 weeks and 6 days of pregnancy; or late preterm - babies born between 34 weeks and 36 weeks and 6 days of pregnancy^7^.

AKI is a common condition in neonatal intensive care units associated with increased mortality^5^^,^ 8. Youssef et al. (2015) reported a frequency of 10.8% for AKI in a neonatal intensive care unit (NICU) and described use of mechanical ventilation and sepsis as the main causes of AKI^9^.

Neonatal sepsis is infection that occurs within the first 28 days of life of a full-term newborn or within four weeks of the predicted date of birth of preterm babies^10^. Neonatal sepsis is a severe complication, particularly for preterm newborns, caused by pathogens acquired after birth. The condition is described as early sepsis for cases with onset within 72 hours of birth or late sepsis for cases developing after 72 hours from birth^10^^,^ 11.

Clinical signs of neonatal sepsis include fever, hypothermia, hypotonia and seizures, irritability and lethargy, difficulty breathing, pallor, gastrointestinal symptoms, idiopathic jaundice, signs of bleeding, and tachycardia^10^^,^ 12.

Preterm birth may adversely affect adaptation to extrauterine life and introduce numerous complications^13^. Factors such as decreased glomerular filtration rate (GFR), renal vasoconstriction, and decreased local blood flow may contribute to the onset of AKI in individuals with sepsis, a condition known for its multifactorial pathophysiology involving hemodynamic and microcirculation mechanisms that ultimately lead to poor tissue oxygenation. Vasoplegia is the primary pathophysiological phenomenon in subjects with septic shock to cause hypotension^14^^-^ 16. Individuals with septic AKI also present with decreased GFR secondary to hypotension and hypovolemia associated with decreased cardiac output, which cause oliguria and increased SCr levels^14^.

Complete blood count is one of the tests used in the investigation of sepsis. Acute phase reactants such as C-reactive protein (CRP) and procalcitonin (PCT) may be used in diagnosis, although blood culture stands as the most recommended test^14^.

In an attempt to standardize the definitions and categories tied to the diagnosis of AKI, the *Risk, Injury, Failure, Loss, End-Stage* (RIFLE) and *Acute Kidney Injury Network* (AKIN) criteria were developed based on parameters SCr and urine output^17^. However, the criteria used to define AKI have since been adjusted, and the most recent version is the *Kidney Disease: Improving Global Outcome* (KDIGO), published in 2012^18^.

Adjustments to account for neonatal populations were made to the criteria in 2013. A study involving the National Institutes of Health, neonatologists, nephrologists, pediatricians, and representatives from the National Institute of Diabetes and Digestive and Kidney Diseases led to the publication of the KDIGO classification for neonates^19^. The KDIGO criteria were updated to better describe AKI in newborns^20^ (Table 1).

**Table 1:** Modified KDIGO (Kidney Disease Improving Global Outcomes) criteria

Stage	Serum creatinine	Urine output
0	No change or increase < 0.3 mg/dL	≥ 0.5 mL/kg/h
1	Increase ≥ 0.3 mg/dL in 48 hours or ≥1.5 to 1.9 times the reference value* ≤ 7 days	< 0.5 mL/kg/h for 6 to 12 hours
2	≥ 2 to 2.9 times the reference value	< 0.5 mL/kg/h for ≥12 hours
3	≥ 3 times the reference value or SCr ≥ 2.5 mg/dL or need for dialysis	< 0.3 mL/kg/h for ≥ 24 hours or anuria for ≥ 12 hours

(*) Reference value: lowest previous creatinine level. SCr: serum creatinine. Source: Adapted from Alconcher et al., 2020^20^.

This review aims to discuss AKI in preterm newborns with sepsis and the workup biomarkers used in the diagnosis, prognosis, and follow-up of individuals with AKI; particular attention was given to non-conventional biomarkers described in recent literature, in order to increase the understanding and awareness of this condition.

## Conventional markers

Although the new criteria for AKI have been validated, diagnosis is still difficult, particularly in newborns. Diagnosis for neonates is based on two functional anomalies: changes in SCr (a marker of GFR) and oliguria, both late markers of kidney involvement^21^.

SCr has limitations concerning renal and non-renal factors. Among renal factors, newborns often present low GFR, and the physiology of the kidneys develops until the age of two years^22^. After birth and depending on gestational age, a newborn's SCr reflects that of its mother; changes (or the absence of change) in SCr might interfere with the diagnosis of AKI; additionally, SCr does not estimate damage, but glomerular function, and may take days to increase after the occurrence of injury^23^.

Interestingly, SCr levels in the general population may not change until 25-50% of kidney function has been lost^21^.

The non-renal factors limiting the use of SCr include age, sex, nutrition, muscle mass, and medication^22^.

Additionally, different methods for determining serum creatinine levels - Jaffe's reaction or the enzymatic method - yield different SCr results^21^.

Limitations still exist in the measurement of urine output, since accuracy depends on the handling of the urinary catheter and a wide range of drugs, particularly diuretics and vasoactive amines^21^.

Biomarker cystatin C (CysC) is a cysteine protease inhibitor synthesized in every nucleated cell in the human body. It works as an endogenous marker of GFR and renal tubular disorder. CysC is freely filtrated in the glomeruli and is completely reabsorbed and not secreted. CysC excretion in urine (uCysC) has been associated with severe acute tubular injury^24^^,^ 25.

A study from China found that serum and urinary CysC levels in patients with sepsis and AKI were higher than the levels seen in patients with sepsis and without AKI^26^.

In patients with AKI, uCysC levels increase after serum CysC levels have increased^27^. Fang et al. (2018) found that uCysC is a sensitive marker of AKI in newborns and a predictor of death^28^.

## Non-traditional biomarkers

Attention has been given to finding the best early diagnostic biomarkers for AKI, so that interventions are developed and outcomes improved^23^. Biomarkers identify normal or pathogenic processes and levels of response to treatment without necessarily being involved in the process of disease, which makes them a valuable tool in the assessment of a patient's condition. They may be used to evaluate proneness to a disease or detect biological anomalies, although they are often used in diagnosis, to measure pathologic conditions, or prognosticate disease development^29^.

Non-traditional biomarkers may be even more useful in assessing response to therapy. Ideally, they should be procured through noninvasive methods (such as urine collection), or with minimum impact on the patients (such as routine blood sampling). Efforts have been made to identify reliable biomarkers of kidney injury in serum, plasma, and urine^29^.

Many are the characteristics of an ideal marker of kidney function, of which the following can be cited: being filtered freely while not binding to macromolecules; not being reabsorbed in the kidneys or secreted by renal tubules; producing reliable GFR estimations with constant production and quick diffusion to extracellular sites; not degrading or being excreted by systems other than the kidneys; being detected and measured by reproducible, accurate laboratory techniques without the interference of other elements; and being affordable^30^.

**Figure 1:**
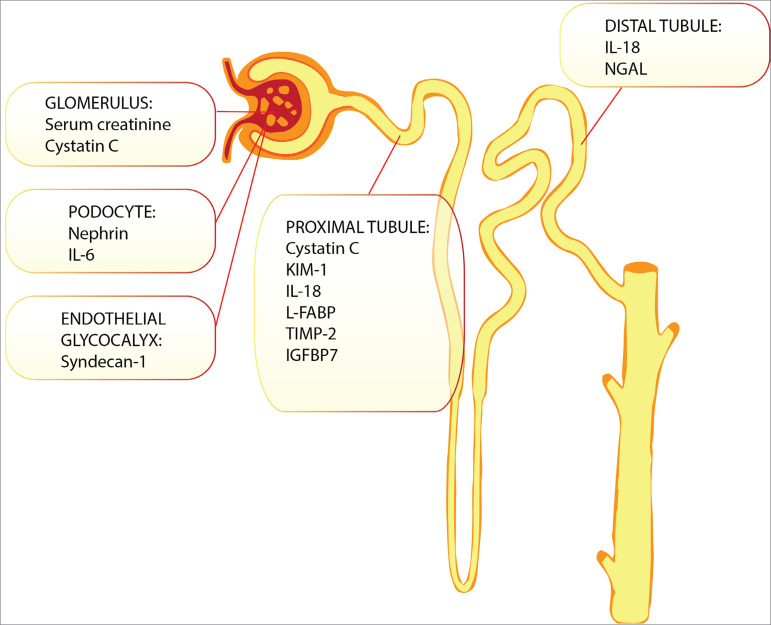
Location of each biomarker of acute kidney injury in the structure of the kidney. Source: Adapted from Malyszko et al., 2015^33^.

Biomarkers may be categorized as follows: inflammatory markers, such as neutrophil gelatinase-associated lipocalin (NGAL), interleukin-6 (IL-6), and interleukin-18 (IL-18); cell damage markers, such as kidney injury molecule-1 (KIM-1) and liver-type fatty acid binding protein (L-FABP); and cell cycle arrest markers, such as tissue inhibitor of metalloproteinases 2 (TIMP2) and insulin-like growth factor-binding protein 7 (IGFBP7)^31^^,^ 32 (Figure 1).

### NGAL

NGAL in its urinary form (uNGAL) originates exclusively from damaged distal nephron epithelial cells, while its serum form (sNGAL) may derive from kidney injury (by tubular leakage) or extrarenal organs interacting with the kidneys^34^^,^ 35. NGAL is a sensitive marker for early detection, accurate prediction, and risk stratification; its levels increase as severity of AKI increases^34^.

Researchers from China published a systematic review and meta-analysis including articles about the use of uNGAL and sNGAL in predicting AKI in patients with sepsis. Their results indicated good diagnostic accuracy for AKI in individuals with sepsis for both marker types^36^.

### IL-6

IL-6 is the most broadly studied cytokine in neonatal populations. It acts on early host response to infection, precedes increases in CRP, and is observed after tumor necrosis factor alpha (TNFα) has been released. IL-6 is produced in endothelial cells, mononuclear phagocytes, fibroblasts, ammonia, trophoblasts, and other cells upon stimulation with microbial products^37^.

IL-6 is a multifunctional cytokine involved in the regulation of immune response and inflammation. It is also known for its proinflammatory activity. IL-6 is one of the elements responsible for the onset and propagation of inflammatory response and is involved in the synthesis of some acute phase proteins (APPs); its levels peak about three hours after injury^38^.

### IL-18

IL-18 is a proinflammatory cytokine produced in proximal tubular epithelial cells in response to injury, to facilitate the synthesis of interferon gamma (IFNγ). After kidney injury, urinary IL-18 (uIL-18) is secreted before there is a significant decrease in kidney function; it is a potential early marker of AKI^39^^,^ 40.

A study enrolling patients with multiple types of kidney disease showed that IL-18 levels were substantially increased in these individuals and that IL-18 was a sensitive and specific marker for acute tubular necrosis (ATN), indicating that it may be a marker of proximal tubular injury in subjects with ATN. The authors also described an association between IL-18 and AKI, since IL-18 levels were significantly increased before SCr increased in patients with acute respiratory failure/acute respiratory distress syndrome who developed AKI, and that IL-18 was a good predictor of death associated with mechanical ventilation^24^.

### KIM-1

Urinary kidney injury molecule-1 (uKIM-1) is a transmembrane glycoprotein not detected in the kidneys or in healthy urine; it is a marker of renal tubule epithelial injury. Its urinary levels are a sensitive predictor of kidney involvement in patients with AKI and may be used as an indicator of poor outcome in the early screening of patients with kidney disease^41^^,^ 42.

A prospective study conducted in China included 150 patients with sepsis and compared, among other parameters, the uKIM-1 levels of survivors and non-survivors with septic AKI. Patients with AKI experienced considerable uKIM-1 increases in six hours, with levels peaking at 24 hours and sustaining until 48 hours after they had been admitted to an ICU^42^. However, in individuals without AKI, KIM-1 remained at baseline levels at various times, similarly to healthy controls (0.85 ± 0.37). Non-survivors had substantially higher levels at 24 and 48 hours, indicating that KIM-1 is a useful early biomarker of septic AKI and that persistent increases in uKIM-1 levels may be associated with poorer outcomes^42^.

### L-FABP

Urinary L-FABP (uL-FABP) is a promising biomarker of tubulointerstitial injury. It is expressed solely in epithelial cells of the proximal tubules in the kidneys. Tubular injury caused by hypoxia increases the synthesis of L-FABP^43^.

A case-control study enrolling 27 pediatric patients submitted to surgery with cardiopulmonary bypass (CPB) described a few remarkable findings: in the group with AKI, SCr levels peaked at 48 h, whereas uL-FABP increased significantly 6 hours after CPB; and L-FABP levels 6 h after CPB were significantly associated with onset of AKI. These findings suggest that L-FABP is a useful biomarker in the early detection of AKI, since it precedes increases in SCr by many hours^44^.

### TIMP-2

TIMP-2 induces cell cycle arrest at the G1 phase, a crucial mechanism in AKI^32^.

This biomarker predicts the development of AKI and has been validated in more than 1,000 critically ill patients for the stratification of risk of injury. It has outperformed other markers and has been considered better for patients with AKI induced by sepsis, although its use has not been approved for individuals aged less than 21 years^32^^,^ 45.

### IGFBP7

IGFBP7 induces cell cycle arrest at the G1 phase in tubular cells in response to injury; it has been associated with AKI^32^^,^ 45.

IGFBP7 is deemed a new biomarker for AKI. It has outperformed other biomarkers in predicting moderate to severe AKI within up to 12 hours of sample collection. IGFBP7 also appears to outperform TIMP-2 in surgery patients. However, its use has not been approved for individuals aged less than 21 years^32^^,^ 45.

### Syndecan-1

Syndecan-1 is a member of the transmembrane proteoglycan family that consistently presents heparan sulfate chains in its structure without cysteine residues^46^. In adult tissues, it is mostly expressed in simple epithelial cells, stratified cells, and plasma cells^47^.

The concept of endothelial injury as an early predictor of AKI was described in cases of leptospirosis, in which syndecan-1 levels were correlated with kidney endothelial glycocalyx damage, a finding known for its association with AKI^48^. A study in which various endothelial biomarkers were analyzed showed that syndecan-1, a biomarker of endothelial glycocalyx dysfunction, was strongly associated with severe AKI in critically ill ICU patients^49^.

### Nephrin

Nephrin is a transmembrane protein expressed in glomerular podocytes. Early podocyte structural alterations are characterized by detachment of podocytes from the glomerular basement membrane. These alterations may produce severe, continuous glomerular injury if the condition persists. Therefore, early recognition of podocyte injury is of great relevance. Urinary nephrin may become an important biomarker of early glomerular injury^50^.

It is unknown whether glomerular injury is induced in the early stages of neonatal AKI. A study with neonates described urinary nephrin as a maturation and glomerular injury biomarker significantly associated with development of AKI and death in NICU settings^51^.

Table 2 shows a summary of the biomarker data presented in this review.

**Table 2:** Origin, pros and cons of acute kidney injury biomarkers

Biomarker	Origin in the kidney	Pros	Cons
Serum creatinine	Glomerulus^21^	SCr is the standard serum marker for the detection of AKI; very inexpensive and offers good chemical stability in clinical practice^24^.	It is a late marker of kidney involvement^21^ affected by age, sex, nutrition, muscle mass, medication^22^, and, after birth, neonatal SCr reflects serum creatinine levels present in the mother^23^.
Cystatin C	Glomeruus^24^ and proximal tubule^52^	It is completely reabsorbed, not secreted, and is not significantly affected by age, sex, ethnicity, or muscle mass^24^.	CysC is not specific enough to distinguish AKI from CKD; its levels increase in a delayed manner; it is considered more as a marker of GFR instead of primary AKI^24^^,^ 40.
NGAL	Distal tubule^34^^,^ 35	Noninvasive; sensitive for accurate early detection; a precise predictor; good for risk stratification; NGAL increases are proportional to the severity of AKI^34^.	Plasma levels include production in organs other than the kidneys; uNGAL is affected by dehydration, difficulty obtaining samples, excessive hydration, and diuretics^30^.
IL-6	Podocytes^53^	This cytokine is a marker of early host infection response; its levels increase before CRP increases; it is produced by numerous cells^37^.	Supporting evidence adjusted to the current KDIGO criteria is still lacking^54^.
IL-18	Proximal^40^ and distal^55^ tubules	IL-18 is a biomarker of ischemic AKI^56^; its levels increase before SCr increases^24^.	Expensive; also derived from myocardial ischemia, lung injury; the entire center may be required to use IL-18 so that cutoff values are established^57^.
KIM-1	Proximal tubule^58^	An early indicator or poor kidney outcome, KIM-1 cannot be detected in healthy kidneys and urine; it is a sensitive predictor of kidney outcome in AKI^41^^,^ 42.	It is used inly in research and presents limitations in the early diagnosis of AKI and recovery monitoring after kidney injury^24^^,^ 40.
L-FABP	Proximal tubule^43^	L-FABP is expressed only in proximal tubule epithelial cells; tubular injury by hypoxia increases the synthesis of this biomarker, making it a promising tool to monitor tubulointerstitial injury^43^.	There is no standardized assay that allows widespread clinical use of L-FABP^40^.
TIMP-2	Proximal tubule^59^	Outperforms all other biomarkers and has produced great results in sepsis-induced AKI^32^.	The current method is time consuming and the biomarker has not been approved for individuals aged less than 21 years; false positive results may lead to unnecessary, expensive testing^45^.
IGFBP7	Proximal tubule^60^	It has outperformed existing biomarkers as a predictor of AKI; it offers great outcomes for surgery patients^32^.	Not approved for individuals aged less than 21 years; false positive results may lead to unnecessary, expensive testing; the current method is time consuming^45^.
Syndecan-1	Endothelial glycocalyx^49^	This biomarker of endothelial glycocalyx injury61 can be used in the diagnostic and prognostic evaluation of sepsis^62^^,^ 63.	Its mechanism in AKI has not been entirely elucidated^64^.
Nephrin	Podocytes^50^	Nephrin may become an important biomarker of early glomerular injury^50^ and AKI in NICU settings, particularly for preterm newborns^51^.	Additional clinical studies are needed to assess and monitor potential uses in the prognostic evaluation of pediatric patients with kidney disease^50^.

## Discussion

The number of reports describing the uses of NGAL in diagnosing AKI has grown steadily. Nga et al. (2015) looked into the development of AKI secondary to sepsis and found that uNGAL was a great predictor of injury within the next 48 hours, with high sensitivity (> 75%) and specificity (> 65%)^65^.

A study enrolling 50 newborns with AKI staying at the NICU of the University Children's Hospital of Skopje, Macedonia, analyzed the incidence, risk factors, and the efficacy NGAL in the early detection of neonatal AKI. The study confirmed the validity of the biomarker in the early diagnosis of AKI in severely ill newborns^66^.

NGAL - in its serum and urinary forms - is a promising marker with favorable results described in the detection and assessment of risk of AKI, which can also be used in cases of sepsis and neonatal AKI.

In terms of cytokines, the literature indicates that serum IL-6 levels may be associated with infection - pneumonia, bacterial peritonitis, and urinary tract infection (UTI) - and death and AKI in patients with liver cirrhosis^67^. In cases of neonatal sepsis, Fan and Yu (2012) did not recommend the isolated use of inflammatory markers CRP, PCT, interleukin-8 (IL-8), TNF-α and interleukin-1 beta (IL-1β); although IL-6 was rated as superior in relation to the majority of markers, it should not be used in isolation^37^.

Greenberg et al. (2015) performed a multicenter study with 106 children aged between one month and 18 years placed on CBP. The authors reported that IL-6 might predict AKI stages 2/3 before surgery and that it was a useful biomarker in scheduling surgery^68^.

Since IL-6 is present since the onset of inflammation, its levels increase earlier than the levels of other biomarkers. IL-6 has been linked to infection, AKI (also in newborns), and possibly neonatal sepsis.

In another study about interleukins, Chinese researchers analyzed 62 severely ill NICU patients without sepsis and showed that IL-18 was a predictor of AKI in this population even after adjustment for gestational age regardless of sex, birth weight, and Apgar score, with the added advantage that it does not decrease as the kidney matures^69^.

Therefore, on account of its presence in inflammation, association with ATN, and role in response to injury, IL-18 increases rapidly and may be seen as a potential biomarker of AKI, including in newborns.

A growing number of studies have investigated the uses of KIM-1 in the diagnosis of neonatal AKI. Genc et al. (2013) studied the uses of uKIM-1 in the early detection of AKI in 48 preterm newborns in intensive care^70^. The authors found a sensitivity of 73.3% and a specificity of 76.9%, and reported that increased uKIM-1 levels on Day 7 were associated with a 7.3-fold increase in the risk of death. The authors concluded that uKIM-1 was a predictor of AKI in neonatal populations^70^.

It is clear that KIM-1 has many uses, ranging from the early diagnosis of AKI to patient prognosis, and that it can be used in neonatal populations and subjects with septic AKI.

In terms of L-FABP, Elnady et al. (2014) carried out a case-control study including 42 newborns with sepsis and AKI in intensive care. Their uL-FABP levels were significantly higher than the levels observed in newborns without AKI^71^.

Significant progress has occurred in research about L-FABP, particularly as the biomarker can be used in the early detection of AKI, since its levels increase before SCr levels increase. Its use has been described in neonatal populations.

In the area of cell cycle arrest biomarkers, Chen et al. (2020) quantified TIMP-2 and IGFBP-7 levels to assess the development of AKI in 237 newborns in intensive care. The combination of the two markers was independently associated with severe AKI, with a sensitivity of 88.9% and a specificity of 50.9%^72^.

Although TIMP-2 has not been approved for subjects aged less than 21 years, studies about its uses in neonatal populations have shown promising results in AKI detection.

IGFBP7 is in a similar situation. Although it has not been approved for neonatal use, studies have described its use in newborns with promising results.

Although the mechanisms tied to sepsis have not been entirely elucidated, it is likely that an association exists between glycocalyx obliteration and sepsis. Increased plasma syndecan-1 levels have been negatively correlated with survival; this significant correlation demonstrates that increased levels of glycocalyx components may be used as diagnostic and prognostic biomarkers in individuals with sepsis^62^^,^ 63.

A prospective cohort study enrolling 289 patients aged less than 18 years and newborns submitted to cardiac surgery at a referral hospital in the state of Ceará, Brazil, showed that early postoperative plasma syndecan-1 levels were independently associated with severe AKI and longer ICU and hospital stays^73^.

The idea of AKI derived from endothelial injury, mainly that associated with endothelial glycocalyx damage, has gained attention and favored new studies involving syndecan-1, especially associated with AKI, including the neonatal population and in sepsis.

Reports have described nephrin as a biomarker of AKI in NICU settings. A study enrolling newborns concluded that initial urinary nephrin levels were higher in newborns with AKI than in newborns without AKI, indicating that increased nephrin levels might occur as a consequence of glomerular immaturity, particularly in preterm newborns^51^.

Considering the development of AKI at a glomerular level, nephrin has collected new indications in health research, leading to relevant findings in neonatal and other populations with AKI.

Conventional and non-conventional biomarkers used in the assessment of AKI present different levels of specificity and sensitivity. Their profiles vary in accordance with the testing and measurement methods, cutoff values, and sample storage protocols^24^. Peres et al. (2013)^24^ recommended a panel of non-conventional biomarkers to diagnose AKI, since each biomarker has its specificities^42^. More studies about biomarkers of AKI for preterm newborns with sepsis are needed, since literature on the subject is still scarce.

As studies have shown, AKI is associated with significant mortality. Early detection allows the introduction of proper treatment and the achievement of better outcomes, in addition to decreasing length of hospitalization, non-medical costs, and morbimortality^5^^,^ 74. In this context, non-traditional biomarkers (such as NGAL, IL-6, IL-18, KIM-1, L-FABP, TIMP-2, IGFBP7, syndecan-1, and nephrin) have a relevant role to play in the early detection of AKI in preterm newborns with sepsis and in the prevention of CKD and death, in addition to mitigating impacts on the healthcare system.

## Conclusion

AKI is a common condition in neonatal intensive care units. It is a multifactorial disease that has sepsis as one of its leading causes, particularly in newborns. AKI is associated with increased mortality. Due to the limitations present in conventional markers, non-traditional biomarkers (such as NGAL, IL-6, IL-18, KIM-1, L-FABP, TIMP-2, IGFBP7, syndecan-1, and nephrin) have become a necessity in the early diagnosis of AKI in newborns with sepsis, so that death rates, length of hospitalization, and the incidence of future complications are decreased.
